# Conformation-based refinement of 18-mer DNA structures

**DOI:** 10.1107/S2059798323004679

**Published:** 2023-06-20

**Authors:** Jakub Svoboda, Daniel Berdár, Petr Kolenko, Jiří Černý, Zora Nováková, Jiří Pavlíček, Bohdan Schneider

**Affiliations:** a Institute of Biotechnology of the Czech Academy of Sciences, BIOCEV, Průmyslová 595, 252 50 Vestec, Czech Republic; b Faculty of Nuclear Sciences and Physical Engineering, Czech Technical University in Prague, Břehová 7, 115 19 Prague 1, Czech Republic; University of Cambridge, United Kingdom

**Keywords:** DNA structure, dnatco.datmos.org, structure validation, structure refinement, base pairing

## Abstract

Nine new crystal structures of CG-rich DNA 18-mers with the sequence 5′-GGTGGGGGC-*XZ*-GCCCCACC-3′, which are related to the bacterial repetitive extragenic palindromes, are reported. 18-mer oligonucleotides with the central *XZ* dinucleotide systematically mutated to all 16 sequences show complex behavior in solution, but all ten so far successfully crystallized 18-mers crystallized as A-form duplexes. The refinement protocol benefited from the recurrent use of geometries of the dinucleotide conformer (NtC) classes as refinement restraints in regions of poor electron density. The restraints are automatically generated at the dnatco.datmos.org web service and are available for download. This NtC-driven protocol significantly helped to stabilize the structure refinement. The NtC-driven refinement protocol can be adapted to other low-resolution data such as cryo-EM maps. To test the quality of the final structural models, a novel validation method based on comparison of the electron density and conformational similarity to the NtC classes was employed.

## Abstract 2

The refinement and validation of nine A-form DNA 18-mer crystal structures containing both canonical and noncanonical base pairs benefits from the use of dinucleotide conformer classes (NtCs).

## 1. Introduction

The ability to form pairs between nitrogenous bases is fundamental to the biological functions of nucleic acids as well as their structural integrity. Base pairs carry genetic information in antiparallel DNA duplexes and are important for establishing the rich 3D architecture of RNA molecules. Genetic code-carrying Watson–Crick (or canonical) base pairs prevail in both DNA and RNA owing their ability to form stable antiparallel self-recognizing duplexes. In genetic DNA duplexes, non-Watson–Crick pairs are often called mis­matches, indicating their potentially malicious effect of miscoding the correct genetic information (Iyer *et al.*, 2006 ▸). In DNA, the formation of non-Watson–Crick pairs influences the duplex geometry by deflecting it from its optimum compatible with the canonical base pairing (Kunz *et al.*, 2009 ▸) and the ability of DNA duplexes to incorporate these pairs depends to a large part on the plasticity of the DNA backbone. The formation of non-Watson–Crick pairs can be stabilized by tautomerism of aromatic rings of the bases that are isosteric with the Watson–Crick pairs and therefore compliant with the helical architecture (Westhof, 2014 ▸).

Duplex destabilization by the formation of non-Watson–Crick pairs increases its structural flexibility, which can lead to the formation of multiple molecular species in solution. Competition of these species in the crystallization batch influences the process of crystallization and may decrease the quality of the resulting crystals or even preclude crystal formation. This fact, and a broader issue of the emergence of DNA and especially RNA structures with low crystallographic resolutions around 3 Å, drew our attention to the process of the refinement of structure models based on lower resolution diffraction data. Refinement protocols for mid- to low-resolution structures need to restrain valence geometric parameters, bond distances and angles, but additional restraining of conformational states helps to improve the quality of the final structural models. The protein structure quality certainly benefits from knowledge of amino-acid rotameric states. In contrast, restraining nucleic acid local conformational states is not part of the standard refinement workflow. This is due to the fact that restraining one or two isolated backbone torsion angles or sugar pucker is a cumbersome and often counterproductive process. Multiple backbone torsions and sugar pucker are correlated, making the nucleic acid conformational space multidimensional. Dinucleotides are the smallest structural fragments that can be classified into well defined classes, so-called dinucleotide conformer (NtC) classes (Černý, Božíková, Svoboda *et al.*, 2020 ▸). The known geometries of the NtC classes provide the possibility to use them as restraints in refinement protocols. The effectiveness of this process needs to be rigorously tested; in this work, we are making the first step.

This paper builds on our previous studies of CG-rich DNA oligonucleotides related to repetitive extragenic palindrome (REP) elements (Charnavets *et al.*, 2015 ▸; Kolenko *et al.*, 2020 ▸), where we have explained the biological relevance of these sequences. The CD spectra of REP-related 18-mers of sequence 5′-GGTGGGGC-*XZ*-GCCCCACC-3′, in which we mutated the central *XZ* dinucleotide, indicated that these 16 oligonucleotides behave differently in solution. Therefore, we wanted to further analyze the structural differences of these 18-mers by X-ray crystallography, especially from the point of view of the differences between *XZ* dinucleotides that are capable and incapable of forming Watson–Crick pairs. We succeeded in the crystallization of nine new 18-mers and present their crystal structures here (Table 1 ▸). The limited quality of the diffraction data of these 18-mers, with resolutions between 2.5 and 3.0 Å, called for a new approach to refinement and we used our knowledge of the NtC classes to restrain the dinucleotide geometries in some of the refined structures. We believe that the modifications to the refinement protocol and the new validation criteria presented here may be beneficial for other lower resolution nucleic acid structures.

## 2. Methods and materials

### 2.1. Crystallization experiments

We studied CG-rich sequences related to bacterial REP elements (Bertels & Rainey, 2011 ▸) from *Cardiobacterium hominis*. Oligonucleotides were synthesized by and purchased from Sigma–Aldrich and Generi Biotech with standard desalting purification.

All crystallized sequences can be written as 5′-GGTGGGGC-*XZ*-GCCCCACC-3′ and we succeeded in crystallization of 18-mers where *XZ* were the dinucleotides AT, AC, AG, CC, CG, GC, GT, TA and TC; they are further named Chom-18-AT, Chom-18-AC, Chom-18-AG *etc.* All oligo­nucleotides were dissolved in 50 m*M* Tris pH 8 to a final concentration of 1 m*M* and stored in the freezer (−18°C). Prior to crystallization, the oligonucleotides were thawed at 20°C, heated to 95°C in a thermoblock for 5 min and cooled to 20°C. Initial screening was performed with the Natrix screen from Hampton Research. The most promising conditions, F2 and F4, were further optimized. Condition F2 consisted of 80 m*M* NaCl (salt), 12 m*M* KCl (salt), 20 m*M* MgCl_2_ (salt), 0.04 *M* sodium cacodylate pH 6.5 (buffer), 30%(*v*/*v*) MPD (precipitant) and 12 m*M* spermine·(HCl)_4_ (additive) and condition F4 consisted of 80 m*M* SrCl_2_ (salt), 0.04 *M* sodium cacodylate pH 6.5 (buffer), 35%(*v*/*v*) MPD (precipitant) and 12 m*M* spermine·(HCl)_4_ (additive). Optimization was performed in a hanging-drop vapor-diffusion setup. The final crystallization conditions are listed in Supplementary Table S1; the volume of the drops was 3 µl, with a 2:1 or 1:1 ratio of DNA stock:reservoir solution, and the reservoir volume was 1000 µl. The variants crystallized within one to four days. Microseeding greatly improved the efficiency of the crystal growth of the Chom-18-AG variant. Crystallization attempts at 10°C failed. Photographs of several crystals are depicted in Supplementary Fig. S1.

The optimized crystallization conditions for all 18-mers contained Sr^2+^ cations. Crystals of several variants initially grew in conditions with a lower concentration of Sr^2+^ or even without the cation, but these conditions produced twinned or small needle-like crystals that were not suitable for diffraction measurements. Further optimization of these conditions that included various metal and nonmetal cations only led to improved crystal quality with solutions containing Sr^2+^ cations. Therefore, we conclude that the interaction of DNA with Sr^2+^ was important for the formation of acceptably well diffracting crystals. We also did not observe the formation of crystals in other conditions without sodium cacodylate, MPD and spermine.

### 2.2. Data collection

Diffraction data were collected at the BESSY II synchrotron operated by the Helmholtz-Zentrum Berlin (Mueller *et al.*, 2015 ▸) and on a D8 Venture (Bruker) diffractometer at the Center of Molecular Structure, Institute of Biotechnology of the Czech Academy of Sciences. Crystals were flash-cooled in liquid nitrogen and data were collected at 100 K. During data collection for Chom-18-AG, we tried lowering the humidity with an HCLab (Arinax). This procedure did not yield better diffraction images. Due to the presence of sufficient amounts of MPD (∼20%) in the crystallization batch, no additional cryoprotective procedure was necessary. Inspection of the diffraction images did not show radiation damage. Mosaicity values were in the range 0.19–0.57°. Diffraction images were processed in *XDS* and *AIMLESS* (Kabsch, 2010 ▸; Agirre *et al.*, 2023 ▸). Raw diffraction images of the best diffracting crystals have been deposited with Zenodo. Data-collection statistics and Zenodo links are given in Table 1 ▸.

### 2.3. Refinement protocol using the NtC classes

*MOLREP* (Vagin & Teplyakov, 2010 ▸; Agirre *et al.*, 2023 ▸) showed that the structure solution for all the 18-mers is almost identical to the structure with PDB code 6ros, with one 18-mer strand in the asymmetric unit (Kolenko *et al.*, 2020 ▸), and we therefore proceeded with rigid-body refinement. Refinement was carried out in an NtC-enhanced local fork of *phenix.refine* version 1.19.2 (Liebschner *et al.*, 2019 ▸); the statistics are listed in Table 2 ▸. Approximately 5% of all reflections were used as a control (free) set.

Despite the involvement of model rebuilding with *Coot* (Emsley *et al.*, 2010 ▸), structure refinement of Chom-18-AT, Chom-18-CG, Chom-18-GC, Chom-18-GT, Chom-18-TC and especially Chom-18-AG remained unstable. Therefore, we decided to restrain the dinucleotide geometries in these structures to the known geometries of the NtC classes. We used the Chom-18-AC variant as the starting reference model because it has the highest resolution and most of its dinucleotides were assigned to NtC classes.

The partial reference model was built from nucleotides 1–7 and 12–18 of Chom-18-AC as described in detail in the following paragraph. The refinement was improved with the aid of NtC-based restraints. The final refinement cycles were performed using all reflections. In the final refinement cycle NtC restraints were kept for steps 1–7 and 12–18. The coordinates and structure factors have been deposited in the PDB (Berman *et al.*, 2002 ▸) and are now available.

Initial model and structure factors were uploaded to the *DNATCO* web service at https://dnatco.datmos.org. After the coordinate file has been uploaded in mmCIF or PDB format, the user is presented with automatically generated NtC restraints for *Phenix* and *CCP*4 (Agirre *et al.*, 2023 ▸) as well as commands for *MacroMolecule Builder* (*MMB*; Flores & Altman, 2010 ▸). The restraints are generated automatically only for model dinucleotides with a root-mean-square deviation (r.m.s.d.) to the closest NtC atoms of within 0.5 Å. The limit of 0.5 Å was determined empirically to restrain only those parts of the structure that are close to the known conformations as defined by the NtC classes. While the default restraints perform well in most cases, the *DNATCO* web service allows finer tuning of the NtC restraint parameters. Users are intuitively guided to choose an alternative NtC based on the provided ‘similarity’ and ‘connectivity’ plots; at the same time, the fit of the newly proposed dinucleotide geometry to the electron density is calculated. Weights of restraint parameters controlling the width (sigma) of the energy function used by the refinement software are assigned automatically. Advanced users can, however, use *DNATCO* to modify the overall weight or even to assign per-dinucleotide weights, allowing tighter control over the refinement. The automatically generated and optional user-tuned restraint files can be downloaded in the Refinement tab under the respective choice of *Phenix*, *REFMAC* or *MMB* software.

NtC restraint files contain the corresponding combinations of torsional and pseudo-bond parameters for the sugar-phosphate backbone torsions, including torsions in the (deoxy)ribose moieties. Below is an excerpt from the *phenix.refine* restraint file for Chom-18-AG: 
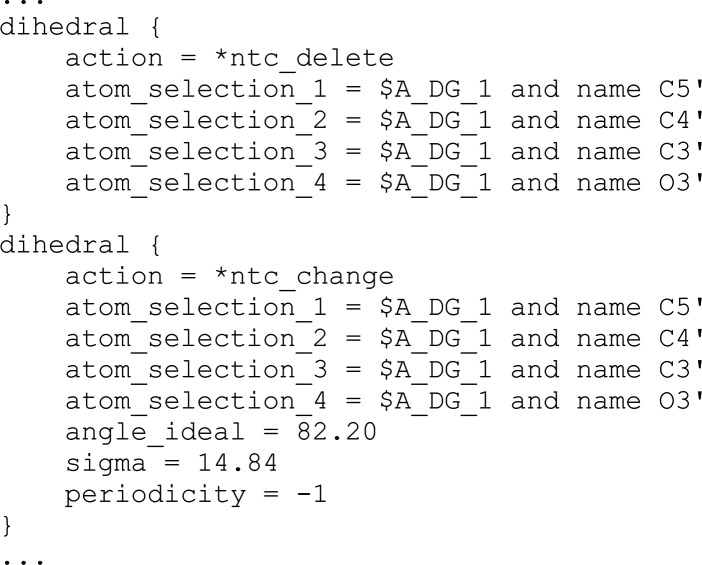


This excerpt modifies one of 22 mostly torsional parameters for the first dinucleotide step in the model. The action keywords ntc_delete and ntc_change are introduced because *phenix.refine* automatically generates a partial set of torsion restraints that are inconsistent with NtC definitions; these restraints are first removed and NtC-derived values are assigned. The base pairs in positions 8–11 were left unrestrained. The restraint file downloaded from dnatco.datmos.org is then edited to add other refinement parameters such as the number of cycles, refinement strategy *etc*. The refinement is then run: phenix.refine coordinates.pdb data.mtz dnatco_refine.params, where dnatco_refine.params is the input file. However, this approach currently requires the patched version of *phenix.refine* available from the *DNATCO* website, which is available upon request.

The restraint file for *REFMAC* works with the current version of the software (Murshudov *et al.*, 2011 ▸). The software was used to independently check the convergence of the refinement process. Below is part of the *REFMAC* restraint file used to refine Chom-18-AG using a tighter sigma for the energy function: 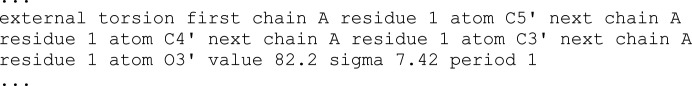


In cases where the selected target NtC differs significantly from the initial model (r.m.s.d. of >0.5 Å as discussed above) or when the patched version of *phenix.refine* is not available, a viable option is to download NtC-related commands for the *MMB* software instead and create a model, which is then used as a reference model in *phenix.refine*. In the case where the actual model geometry and the geometry targeted by the restraints are distant, the target values can be omitted from the refinement. Larger rearrangements of the model using only NtC restraints can thus fail and *MMB* or *Coot* intervention or the use of the ‘idealized’ model from *DNATCO* is needed.

In summary, the NtC classes guide the refinement procedure from the initial conformation to the final model. Section 3.1 describes the practical steps of refinement of Chom-18-AG (PDB entry 7z82), the structure with the worst quality diffraction, Chom-18-TC (PDB entry 7z81) and Chom-18-CG (PDB entry 7z7u).

## 3. Results and discussion

### 3.1. NtC-driven refinement

NtC classes provided valuable help with building the initial models of six of the nine 18-mers, *i.e.* Chom-18-AT, Chom-18-CG, Chom-18-GC, Chom-18-GT, Chom-18-TC and Chom-18-AG. The rationale for using the geometries of the NtC classes as refinement restraints is that NtC classes, which are defined by the most probable combinations of 12 sugar-phosphate backbone geometric parameters, represent the most probable dinucleotide structures (Černý, Božíková, Svoboda *et al.*, 2020 ▸). In a broader context, NtC classes correspond to dinucleotide local energy minima. It is therefore logical to use them as guides for fitting and refining low-resolution electron densities, where the stress on parametrization of refinement is more consequential.

The NtC-supported refinement was most useful in the case of Chom-18-AG with a dipurinic base pair (PDB entry 7z82). The crystals of Chom-18-AG diffracted to the lowest crystallographic resolution of ∼3 Å. The refinement was initially unstable, producing several negative peaks along the sugar-phosphate backbone in the map and unsatisfactory *R*_work_ and *R*_free_ values of 0.305 and 0.431, respectively. To improve the results of refinement, we generated the NtC restraints, which provided torsional refinement parameters for *phenix.refine*. This decreased the *R*_work_ and *R*_free_ values to final values of 0.297 and 0.322, respectively (Table 2 ▸). In addition to this improvement, we also noticed cleaner electron-density maps with fewer diffraction minima along the sugar-phosphate backbone compared with the starting phases of the refinement cycle. As expected, closeness to the NtC standards increased substantially. The average confal score (the confal score quantifies the agreement between the analyzed dinucleotides and the NtC-defining conformers; for details, see Schneider *et al.*, 2018 ▸) for the entire structure increased significantly from 46 to 64, corresponding to a shift from the 47th to the 84th percentile with respect to all nucleic acid structures in the PDB. The number of unassigned (NANT) dinucleotide steps changed from five to four (Table 3 ▸ and Supplementary Table S2).

**Figure 1:**
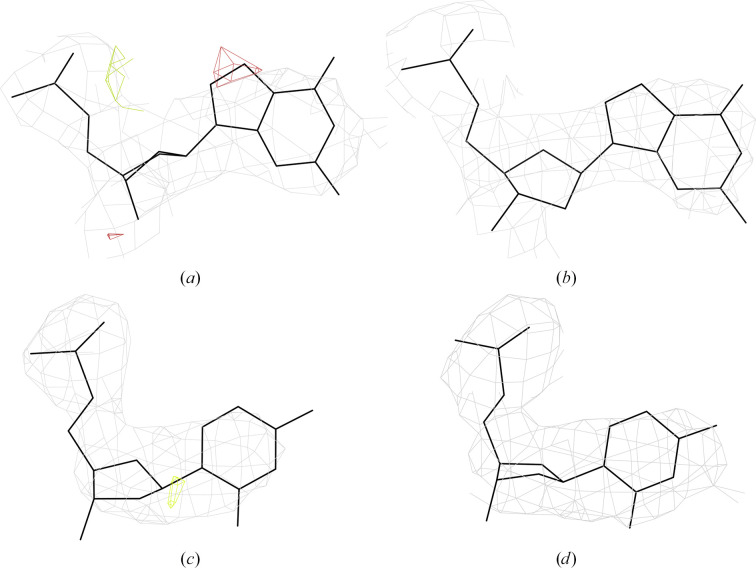
Comparison of NtC-nonrestrained (*a*, *c*) and restrained (*b*, *d*) refinement of residue DG4 of Chom-18-CG (*a*, *b*) and residue DC18 of Chom-18-TC (*c*, *d*). The 2*mF*_o_ − *DF*_c_ electron density is contoured in gray at the 1σ level and the *mF*_o_ − *DF*_c_ electron density is contoured in green for positive and in red for negative at the 3σ level. Images were drawn with *CCP*4*MG* (McNicholas *et al.*, 2011 ▸).

While the NtC-unrestrained Chom-18-CG model had two dinucleotides that were unassigned to NtC classes, all di­nucleotides are assigned to NtC classes in the restrained model (Table 3 ▸ and Supplementary Table S2). Although the number of unassigned steps remained the same in the Chom-18-TC structure, the average confal score and the average RSCC improved. The r.m.s.d.s between the NtC-restrained and nonrestrained models were 0.45 and 0.57 Å for the Chom-18-TC and Chom-18-CG structures, respectively (Table 3 ▸). Additionally, fewer sessions and cycles of refinement and manual rebuilding were necessary compared with refinement unrestrained by NtCs. Application of the NtC geometrical restraints improved the fit to the electron density marginally; the RSCCs of the constrained and nonconstrained models remained approximately the same (Table 3 ▸ and Fig. 1 ▸). In the case of the other structures, the use of NtCs decreased the *R*_work_ and *R*_free_ values marginally, but the geometrical closeness to the NtC classes increased (Supplementary Table S2).

To summarize, our first experience with NtC-restrained refinement indicates that it makes the refinement process more robust for lower quality diffraction data and improves the fit to the electron density, and at the same time improves the agreement with the known conformations, as represented here by dinucleotide NtC fragments.

### 3.2. General features of the 18-mer DNA structures

**Figure 2:**
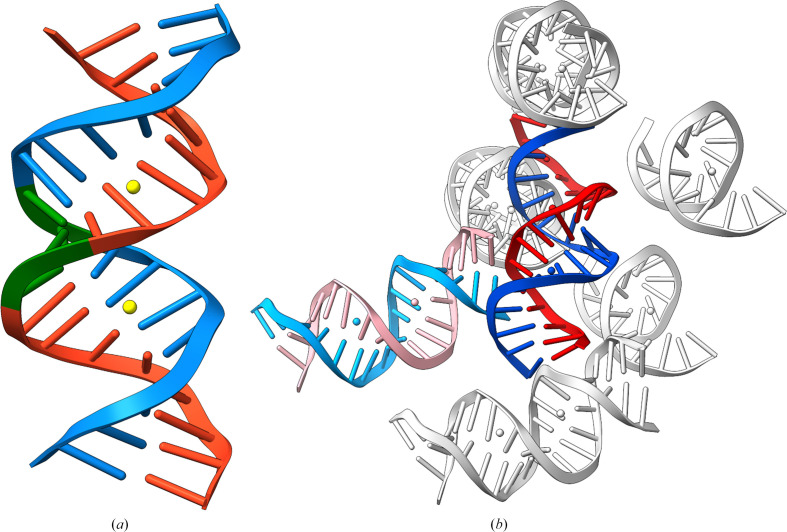
The architecture and crystal packing of ten analyzed DNA 18-mers. (*a*) The duplexes have the overall shape of the A-form. The two symmetry-related strands are colored blue and red, the two central nucleotides are depicted in green and the yellow spheres are Sr^2+^ cations. (*b*) The crystal packing. Two duplexes whose atoms are closer than 4.0 Å are highlighted in red and blue; all duplexes in gray are further than 4.0 Å from these two duplexes. Images were drawn for Chom-18-AC (PDB entry 7z7l) using *ChimeraX* (Pettersen *et al.*, 2021 ▸).

All 18-mers crystallized as isomorphic tetragonal crystals with one strand of a right-handed antiparallel duplex in the asymmetric unit. Pictures of electron densities are given in Supplementary Table S2. The structures can be characterized overall as deformed A-form duplexes (Fig. 2 ▸ *a*). Four of the new structures describe palindromic duplexes potentially with all Watson–Crick pairs: Chom-18-AT (PDB entry 7z7k), Chom-18-CG (PDB entry 7z7u), Chom-18-GC (PDB entry 7z7w) and Chom-18-TA (PDB entry 7z7z). Six 18-mers have sequences with the two central nucleotides forming non-Watson–Crick pairs: Chom-18-AC (PDB entry 7z7l), Chom-18-CC (PDB entry 7z7m), Chom-18-GT (PDB entry 7z7y), Chom-18-TC (PDB entry 7z81), Chom-18-TT (PDB entry 6ros; Kolenko, Svoboda *et al.* 2020 ▸) and Chom-18-AG (PDB entry 7z82). Detailed analysis of base pairing and backbone geometry is provided below.

The crystal packing of all structures is virtually identical. The strand in the asymmetric unit forms the duplex by base-pairing with the other strand related by the twofold axis. The duplexes are only weakly connected. In each structure there are fewer than 30 unique DNA–DNA contacts shorter than 4 Å. The contacts occur between the base atoms of one duplex and the deoxyribose and phosphate atoms of a symmetry-related duplex outside the mutated central region. The list of contacts for Chom-18-AC (PDB entry 7z7l) is given in Supplementary Table S3; the smallest number of contacts shorter than 4.0 Å (21) is observed in Chom-18-GT (PDB entry 7z7y) and the largest number (28) is observed in Chom-18-AC (PDB entry 7z7l). The touching duplexes are highlighted in color in Fig. 2 ▸(*b*). The two central variable dinucleotides do not directly participate in crystal packing; the distances of their atoms to the atoms of symmetry-related duplexes are greater than 6.5 Å. As we have already discussed (Kolenko *et al.*, 2020 ▸), this packing arrangement is reminiscent of that observed in octamers, for instance d(GGGGCCCC)_2_ (PDB entry 2ana; McCall *et al.*, 1985 ▸), and in d(GCGGGCCCGC)_2_ decamers (PDB entries 137d and 138d; Rama­krishnan & Sundaralingam, 1993 ▸), where two neighboring sugar rings of one strand stack on the first base pair of a symmetry-related duplex. In all three cited cases, the hydrophobic surfaces of the terminal base pairs stack on the sugar ring edges and may form a few direct or water-bridged (PDB entries 136d and 137d) hydrogen bonds. It is notable that similar packing interactions occur for duplexes of different lengths of eight, ten and 18 nucleotides. All of these duplexes crystallized in different space groups.

All ten analyzed structures have most of the dinucleotides in A-like conformers. The AA00 class describing the canonical A-form prevails, while the less populated A-like NtC classes (AA08, AA04 and AA01) occur more in the central region (Table 4 ▸). Only Chom-18-CG, Chom-18-GC and Chom-18-AC have all dinucleotides assigned (classified as NtC classes AA##); unassigned dinucleotides (NtC class NANT) are mostly localized near the central base pair. Most dinucleotides with deformed backbones and unassigned dinucleotides are observed in Chom-18-YY and especially Chom-18-AG, pointing to a highly deformed backbone.

Despite the overall similarity of the duplexes, the central region with a variable dinucleotide sequence shows a trend depending on the central dinucleotide. When we measure the distances between the C1′ atom of nucleotide 9 and C1′ of its symmetry-related base-paired nucleotide 10 in all 18-mers, the order from the shortest to the longest is TT (7.9 Å) < CC < TC < GT < GC < AC < AT < TA < CG < AG (12.1 Å). This trend follows the size of the pyrimidine–pyrimidine (Y–Y), pyrimidine–purine (Y–R/R–Y) and purine–purine (R–R) pairs regardless of the type of base pair involved. The same pattern is observed for P–P distances across the strand (data not shown). The poor quality of the Chom-18-AG crystals and the unsuccessful crystallization of the three R–R 18-mers with central GG, GA and AA dinucleotides may indicate that the central pairs of these R–R 18-mers are becoming too large to be accommodated in the same helical architecture. The observation that the crystal packing can accommodate relatively small changes in the molecular shape has been made previously on a set of Dickerson–Drew dodecamer structures (Dickerson *et al.*, 1994 ▸).

All reported structures co-crystallized with the Sr^2+^ cation located between nucleotides 6 and 7 and (by symmetry) 12 and 13. Sr^2+^ cations interact with the keto O6 atoms of guanines 6 and 7. The second Sr^2+^ cation is observed in Chom-18-GT and Chom-18-TT (PDB entries 7z7y and 6ros, respectively). Chom-18-TT also contains a third Sr^2+^ cation observed at the twofold axis between the central pairs 9 and 10.

As in our previous studies of REP-related oligonucleotides (Charnavets *et al.*, 2015 ▸; Kolenko *et al.*, 2020 ▸), we investigated the behavior of the DNA in solution by circular dichroism. The spectra of all ten analyzed 18-mers show complex sequence-dependent features that are described in the supporting information and Supplementary Fig. S3.

### 3.3. Validation by correlation between electron density and geometry

The annotation of nucleic acid structures by NtC classes opens a way to a simple yet powerful validation of the structure quality by correlating the geometries of analyzed dinucleotides and their fit to the experimental electron density. For each dinucleotide, we performed the following.

(i) We compared the geometry of the model with the geometries of dinucleotides in the curated ensemble of dinucleotides with defined geometries, the so-called ‘golden set’ defining the NtC classes; the similarity is measured as the r.m.s.d. in Cartesian space (Černý, Božíková, Svoboda *et al.*, 2020 ▸). These r.m.s.d. values, which are calculated for both assigned and unassigned dinucleotides, gauge the geometric similarity between analyzed dinucleotides and dinucleotides in the golden set.
(ii) We calculated the real-space correlation coefficient (RSCC) of electron densities of the model and experiment. Electron densities were calculated using *phenix.real_space_refine* from all atoms in the step and the resulting RSCC as a mean average from individual atomic correlations (Afonine *et al.*, 2018 ▸).
(iii) We display the calculated RSCC and r.m.s.d. values as scattergrams that plot values for individual dinucleotides as points or for an ensemble of structures as contour plots.

**Figure 3:**
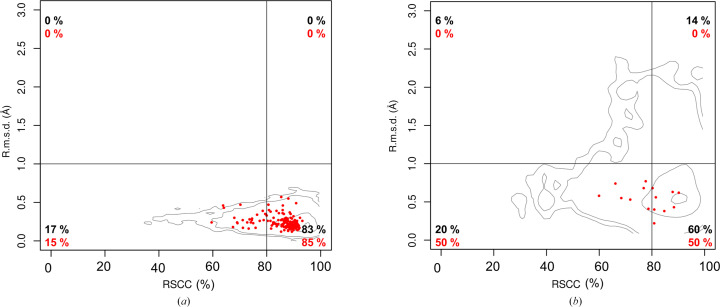
Scattergrams of real-space correlation coefficients (RSCCs) and root-mean-square deviations (r.m.s.d.s) of dinucleotides that are (*a*) assigned and (*b*) unassigned to NtC classes. The gray contours denote values for 99%, 95%, 50% and 5% of values in the data set of a curated ensemble of 497 chains of sequentially nonredundant and uncomplexed DNA from previously selected crystal structures with crystallographic resolution higher than 2.6 Å (Biedermannová *et al.*, 2022 ▸). The red dots mark the values for dinucleotides of ten analyzed structures. Vertical and horizontal lines represent borders between values that are deemed to be acceptable and poor. Details of the protocol for calculating the RSCC and r.m.s.d. values are given in the text and in Černý, Božíková, Svoboda *et al.* (2020 ▸).

Fig. 3 ▸ displays the RSCC–r.m.s.d. scattergrams calculated for dinucleotides of ten analyzed structures (red dots) and, as gray contours, values calculated for a curated ensemble of 497 chains of sequentially nonredundant uncomplexed DNA from crystal structures with crystallographic resolution higher than 2.6 Å selected according to Biedermannová *et al.* (2022 ▸).

In Fig. 3 ▸, we show two scattergrams, the first displaying the relationship between RSCC and r.m.s.d. for all dinucleotides assigned to NtC classes and the second displaying the same relationship for unassigned dinucleotides (formally class NANT). The data in the pictures are divided into four rectangles by the vertical line separating dinucleotides whose model and experimental electron densities correlate at 80% and the horizontal line for r.m.s.d. values of 1.0 Å. The data in the rectangles are interpreted as follows.

(i) Lower right: ‘good’ dinucleotides with known geometry and a good fit to electron density.
(ii) Lower left: ‘over-refined’ dinucleotides with known geometry and a poor fit to electron density.
(iii) Upper right: ‘unique’ dinucleotides with unknown geometry and a good fit to electron density.
(iv) Upper left: ‘poor’ dinucleotides with unknown geometry and a poor fit to electron density.

The difference between the scattergrams for assigned and unassigned dinucleotides is evident. The assigned dinucleotides (Fig. 3 ▸ *a*) have a large majority of dinucleotides in rectangle (i) (‘good’ structures), but a significant fraction of dinucleotides are still ‘over-refined’ in rectangle (ii). The distributions of the template ensemble (gray contours) and the analyzed structures (red dots) are about the same. A large fraction of over-refined dinucleotides can be interpreted as the fitting of geometrically well known fragments into inconclusively shaped electron density.

In contrast, the RSCC–r.m.s.d. scattergram looks different for unassigned dinucleotides (Fig. 3 ▸ *b*). The values of the reference ensemble of structures are scattered in all four rectangles, with significant fractions of over-refined (20%), unique (14%) and even poor (6%) dinucleotide geometries. The unassigned dinucleotides from ten analyzed structures are distributed evenly between the good and over-refined rectangles. The distributions of the reference and analyzed dinucleotides are different because the underlying structures are different: while the reference set contains variable structures with potentially uniquely shaped dinucleotides [upper right quadrant (iii)], the dinucleotides in the analyzed structures are all part of conventional double helices that do not depart from conventional A-like conformations close to the NtC classes AA##. In such a case, refinement does not call for a radical departure from the known conformations and converges in the over-refined quadrant (ii).

### 3.4. Base pairing

**Figure 4:**
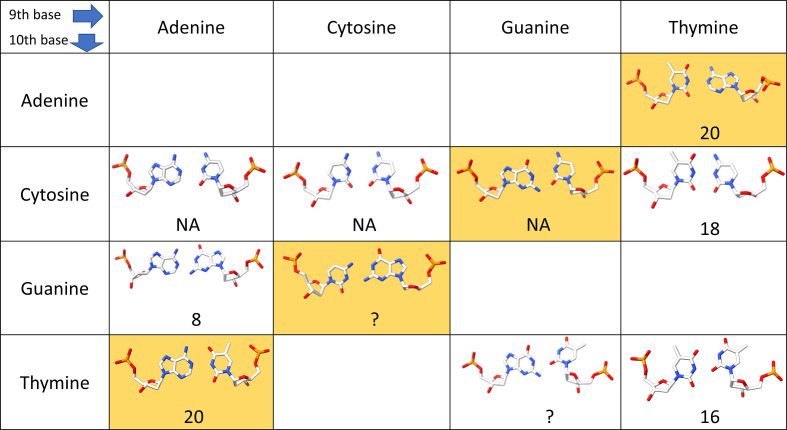
Topologies of the central base pairs (residue 9 and symmetry-related residue 10) in ten analyzed structures. The numbers in the insets indicate the Saenger base-pair notation (Saenger, 1984 ▸) assigned by the PDB during deposition. Steps labeled ‘?’ have the base-pair type unassigned; no base-pairing information was provided for the pairs labeled NA. We highlight potential Watson–Crick pairs in yellow.

All central base pairs in the ten analyzed structures form base pairs by Watson–Crick edges (Leontis & Westhof, 2001 ▸). Fig. 4 ▸ summarizes the assignments of these base pairs in the Saenger notation (Saenger, 1984 ▸) as archived by the PDB in mmCIF files as _ndb_struct_na_base_pair.hbond_type_28.

#### 3.4.1. 18-mers with all nucleotides able to form Watson–Crick pairs

All four Chom-18-mers with two central nucleotides (residues 9 and 10) able to form Watson–Crick pairs were crystallized. Base pairs A–T and T–A are classified as Watson–Crick pairs, while the C–G pair adopts a specific orientation characterized by a large value of one of the base parameters, shear (2.97 Å), and is not classified. The topology of the G–C pair is compatible with the Watson–Crick pair, but it was not classified as a pair because its atoms do not comply with the hydrogen-bond geometry.

#### 3.4.2. 18-mers with two central nucleotides not able to form canonical base pairs

Of the four YY 18-mers, only Chom-18-CT could not be crystallized. Both Chom-18-TC and Chom-18-CC have high propeller twist; its extreme value in Chom-18-CC precludes assignment of the base-pair category. The geometry of the base pair in Chom-18-TT is different due to the interaction of the thymine O4 major-groove O atoms with the Sr^2+^ cation.

Two of the four YR and RY variants unable to form Watson–Crick pairs were crystallized, Chom-18-AC and Chom-18-GT, but their base-pairing topology was not assigned.

Finally, only one of the four RR variants, Chom-18-AG, was crystallized. The A–G pair is strongly nonplanar; despite this, the pair is classified. Two successive voluminous A–G base ‘pairs’ were observed in a decamer crystal structure (PDB entry 1d8x; Gao *et al.*, 1999 ▸). In analogy to Chom-18-AG, the bases of PDB entry 1d8x are moved from their common plane; this effect is called ‘sheared bases’ in the original paper.

Structures of Chom-18-mers with central dinucleotides that are capable and incapable of forming Watson–Crick pairs are not distinguishable by any single geometrically interpretable feature of the backbone such as NtC class (Table 4 ▸ and Supplementary Table S4) or base parameters (supporting information and Supplementary Fig. S4); their backbone geometries are locked in the A-form duplex (Fig. 2 ▸).

## 4. Conclusions

Of the 16 permutations of the central dinucleotide in 18-mer oligonucleotides 5′-GGTGGGGC-*XZ*-GCCCCACC-3′, we crystallized ten. The possible *XZ* combinations are indicated in Fig. 4 ▸. Nine structures are reported here (Tables 1 ▸ and 2 ▸) and we analyze them together with our previously reported structure with PDB code 6ros (Kolenko *et al.*, 2020 ▸). All oligonucleotides crystallized as isomorphic A-form duplexes (Fig. 2 ▸) despite their circular-dichroism spectra showing complex structural behavior, which is likely to be caused by conformational heterogeneity in solution.

The diffraction data for the analyzed structures were of limited resolution between 2.5 and 3.0 Å and the refinement of six newly determined structures was not stable. Restraining the dinucleotide geometries by the geometries of the dinucleotide conformer (NtC) classes (Černý, Božíková, Svoboda et al., 2020 ▸) improved the convergence of the refinement, improved the fit to the electron density and decreased the *R*_free_ values. The restraints are automatically generated by the dnatco.datmos.org web service and are available for download. The refinement protocol benefited significantly from the recurrent use of geometries of the NtC classes as restraints because it stabilized the final models especially in regions of diffuse electron density. The proposed protocol is quite general and is generalizable to other crystal structures. Its applicability to cryo-EM data of nucleic acid structures needs to be tested.

The structures of Chom-18-mers with a central dinucleotide capable and incapable of forming Watson–Crick pairs are not distinguishable by any single geometrically interpretable feature. The local geometric distortions from the A-form as described by the NtC classes are not reflected immediately at the central mismatched nucleotides but they propagate in the direction of the strand (Table 3 ▸, Supplementary Fig. S4).

To validate structural qualities, we employed our previously developed analysis using two-dimensional scattergrams of RSCC and r.m.s.d. values (Černý, Božíková, Svoboda *et al.* 2020 ▸; Fig. 3 ▸). The scattergrams provide an easy visual indication of potentially incorrectly refined structural fragments and thus help in quick validation regardless of the size and complexity of the structure.

## 5. Data availability

The presented data are available from the Protein Data Bank as PDB entries 7z7l (Chom-18-AC), 7z82 (Chom-18-AG), 7z7k (Chom-18-AT), 7z7m (Chom-18-CC), 7z7u (Chom-18-CG), 7z7w (Chom-18-GC), 7z7y (Chom-18-GT), 7z7z (Chom-18-TA and 7z81 (Chom-18-TC). Diffraction images have been deposited with the Zenodo server (see Table 1 ▸).

## 6. Related literature

The following references are cited in the supporting information for this article: Hoogsteen (1963 ▸), Jaumot *et al.* (2002 ▸), Kim *et al.* (1993 ▸), Li *et al.* (2019 ▸), Neidle (2008 ▸), Nikolova *et al.* (2011 ▸), Skelly *et al.* (1993 ▸), del Villar-Guerra *et al.* (2018 ▸) and Vorlíčková *et al.* (2012 ▸).

## Supplementary Material

PDB reference: Chom-18-AT, 7z7k

PDB reference: Chom-18-AC, 7z7l

PDB reference: Chom-18-CC, 7z7m

PDB reference: Chom-18-CG, 7z7u

PDB reference: Chom-18-GC, 7z7w

PDB reference: Chom-18-GT, 7z7y

PDB reference: Chom-18-TA, 7z7z

PDB reference: Chom-18-TC, 7z81

PDB reference: Chom-18-AG, 7z82

Raw diffraction images for PDB entry 7z7k.: https://10.5281/zenodo.6333817

Raw diffraction images for PDB entry 7z7l.: https://10.5281/zenodo.6336683

Raw diffraction images for PDB entry 7z7m.: https://10.5281/zenodo.6336722

Raw diffraction images for PDB entry 7z7u.: https://10.5281/zenodo.6336839

Raw diffraction images for PDB entry 7z7w.: https://10.5281/zenodo.6337128

Raw diffraction images for PDB entry 7z7y.: https://10.5281/zenodo.6597387

Raw diffraction images for PDB entry 7z7z.: https://10.5281/zenodo.6597824

Raw diffraction images for PDB entry 7z81.: https://10.5281/zenodo.6336683

Raw diffraction images for PDB entry 7z82.: https://10.5281/zenodo.6336707

Supplementary text and Supplementary Tables and Figures. DOI: 10.1107/S2059798323004679/rr5230sup1.pdf

## Figures and Tables

**Table:** Values in parentheses are for the outer shell. All 18-mers produced isomorphous tetragonal crystals belonging to space group *P*4_3_2_1_2. They were measured at 100 K either on BESSY beamline 14.1 or by using a Bruker Venture D8 with a liquid-jet anode at the Center of Molecular Structure at the Institute of Biotechnology, Czech Academy of Sciences (IBT).

*XZ*	AC	AG	AT	CC	CG
PDB code	7z7l	7z82	7z7k	7z7m	7z7u
Diffraction source	BESSY	IBT	BESSY	IBT	BESSY
Wavelength (Å)	0.9184	1.3418	0.9184	1.3418	0.9184
Rotation range per image	0.1	0.5	0.1	0.5	0.1
Exposure time per image (s)	0.1	90	0.1	70	0.1
*a* = *b* (Å)	37.76	38.11	38.19	38.09	38.28
*c* (Å)	85.11	87.81	87.82	87.42	87.32
Resolution range (Å)	22.68–2.50 (2.59–2.50)	34.96–2.95 (3.19–2.95)	43.91–2.70 (2.85–2.70)	28.72–2.40 (2.50–2.40)	43.66–2.75 (2.92–2.75)
Total No. of reflections	60497 (7806)	48048 (8569)	45763 (6957)	86942 (10007)	44663 (7411)
Unique reflections	2563 (298)	1535 (291)	2062 (280)	2658 (301)	1958 (297)
Completeness (%)	99.9 (99.8)	97.9 (93.6)	100 (100)	94.4 (99.3)	100 (100)
Multiplicity	23.6 (26.2)	31.3 (29.4)	22.2 (24.8)	32.7 (33.2)	22.8 (25.0)
〈*I*/σ(*I*)〉	25.7 (2.8)	35.8 (4.1)	22.6 (0.9)	35.7 (2.9)	29.4 (1.9)
CC_1/2_	0.997 (0.910)	0.999 (0.962)	0.999 (0.678)	0.998 (0.992)	0.999 (0.895)
CC*	0.999 (0.976)	0.996 (0.990)	0.997 (0.899)	0.997 (0.998)	0.998 (0.972)
Data availability	https://10.5281/zenodo.6336683	https://10.5281/zenodo.6336707	https://10.5281/zenodo.6333817	https://10.5281/zenodo.6336722	https://10.5281/zenodo.6336839

**Table:** 

*XZ*	GC	GT	TA	TC
PDB code	7z7w	7z7y	7z7z	7z81
Diffraction source	BESSY	BESSY	IBT	BESSY
Wavelength (Å)	0.9184	0.9184	1.3418	0.9184
Rotation range per image	0.1	0.1	0.5	0.1
Exposure time per image (s)	0.1	0.1	30	0.1
*a* = *b* (Å)	38.62	37.70	38.48	38.20
*c* (Å)	87.95	89.72	90.33	88.38
Resolution range (Å)	43.97–2.75 (2.92–2.75)	44.86–2.50 (2.61–2.50)	45.17–2.60 (2.73–2.60)	44.19–2.75 (2.92–2.75)
Total No. of reflections	45103 (7363)	59296 (7357)	27067 (3600)	44499 (7534)
Unique reflections	1997 (295)	2556 (288)	2394 (308)	1971 (304)
Completeness (%)	99.9 (99.6)	99.9 (100)	100 (100)	100 (100)
Multiplicity	22.6 (25.0)	23.2 (25.5)	11.3 (11.7)	22.6 (24.8)
〈*I*/σ(*I*)〉	25.3 (2.1)	31.2 (4.7)	15.2 (2.0)	26.4 (2.1)
CC_1/2_	0.999 (0.908)	0.996 (0.908)	0.999 (0.618)	0.988 (0.915)
CC*	0.993 (0.976)	0.996 (0.976)	0.995 (0.874)	0.977 (0.978)
Data availability	https://10.5281/zenodo.6337128	https://10.5281/zenodo.6597387	https://10.5281/zenodo.6597824	https://10.5281/zenodo.6598165

**Table 2:** Refinement statistics for DNA 18-mers with sequence 5′-GGTGGGGC-*XZ*-GCCCCACC-3′ The diffraction precision index (DPI) was calculated at https://cluster.physics.iisc.ernet.in/dpi/ (Kumar *et al.*, 2015 ▸).

*XZ*	AC	AG	AT	CC	CG	GC	GT	TA	TC
PDB code	7z7l	7z82	7z7k	7z7m	7z7u	7z7w	7z7y	7z7z	7z81
No. of reflections, working set	2396	1230	1994	2092	1822	1952	2027	1731	1841
No. of reflections, test set	138	51	105	88	106	110	114	94	97
*R*_work_	0.241	0.297	0.261	0.233	0.274	0.258	0.258	0.264	0.273
*R*_free_	0.331	0.322	0.336	0.292	0.289	0.333	0.319	0.308	0.338
*R*_all_	0.252	0.299	0.271	0.238	0.283	0.266	0.272	0.269	0.277
No. of non-H atoms									
DNA	365	368	366	363	366	366	367	366	364
Ions	1	1	1	1	1	1	2	1	1
Total	366	369	367	364	367	367	369	367	365
R.m.s. deviations									
Bond lengths (Å)	0.005	0.011	0.011	0.011	0.008	0.008	0.01	0.009	0.006
Angles (°)	0.492	1.276	1.648	1.277	0.718	0.747	1.014	0.826	0.745
Average *B* factors (Å^2^)	89	117	93	67	105	96	84	102	109
DPI	0.379	0.567	0.592	0.470	0.673	0.619	0.570	0.905	0.735

**Table 3:** Comparison of the model quality obtained using refinement protocols without and with NtC restraints for Chom-18-TC, Chom-18-CG and Chom-18-AG The table lists changes in the confal score (Δconfal) and RSCC (ΔRSCC) of the restrained − nonrestrained models. When the NtC restraints were used, *R*_free_ decreased for these three structures by 1.9%, 2.0% and 12.3%, respectively. The overall r.m.s.d.s between the NtC-restrained and nonrestrained models are 0.45, 0.57 and 1.36 Å, respectively. Supplementary Table S2 lists similar values with more details for all structures that were restrained with the NtC-based parameters.

	Chom-18-TC (PDB entry 7z81)		Chom-18-CG (PDB entry 7z7u)		Chom-18-AG (PDB entry 7z82)	
Step	Δconfal	ΔRSCC	Δconfal	ΔRSCC	Δconfal	ΔRSCC
1–2	53	0.001	−9	−0.020	17	0.021
2–3	46	−0.005	3	−0.014	9	0.016
3–4	16	0.005	5	−0.031	18	0.007
4–5	32	0.023	82	−0.035	79	0.013
5–6	22	0.045	38	−0.014	7	0.012
6–7	9	0.097	17	−0.002	49	0.002
7–8	9	0.070	4	0.012	−9	0.010
8–9	50	0.011	−26	0.004	19	0.000
9–10	0	0.002	10	0.001	−27	0.018
10–11	−9	−0.001	47	0.051	0	0.009
11–12	0	−0.014	25	0.059	0	−0.042
12–13	−16	−0.020	−11	−0.011	56	−0.027
13–14	21	0.016	31	−0.020	20	−0.018
14–15	0	0.023	−26	−0.008	−3	−0.017
15–16	−9	0.010	−22	−0.018	−21	0.004
16–17	4	0.003	−2	−0.003	26	0.025
17–18	85	−0.002	−5	−0.012	86	0.037
Overall	18	0.014	9	0.011	18	0.065

**Table 4:** NtC classes for the central regions of ten analyzed DNA 18-mers with sequence 5′-GGTGGGGC-*XZ*-GCCCCACC-3′ A table of the NtC assignments for all dinucleotides is provided as Supplementary Table S4. The NtC assignments can also be analyzed in greater detail at the website https://dnatco.datmos.org (Černý, Božíková, Maly *et al.*, 2020 ▸; Černý, Božíková, Svoboda *et al.* 2020 ▸).

*XZ*	AT	CG	GC	TA	CC	TC	TT	AC	GT	AG
PDB code	7z7k	7z7u	7z7w	7z7z	7z7m	7z81	6ros	7z7l	7z7y	7z82
7–8	AA08	AA08	AA08	NANT	AA08	AA08	AA08	AA08	NANT	NANT
8–9	AA00	AA11	AA00	AA08	AA00	AA00	AA00	AA00	AA00	AB01
9–10	AA03	AA08	AA08	NANT	AA00	AA00	AA08	AA00	AA00	NANT
10–11	NANT	AA00	AA00	AA00	NANT	NANT	NANT	AA06	AA06	NANT
11–12	AA01	AA10	AA10	AA08	NANT	NANT	NANT	AA11	AA11	NANT
