# Supplementary material for: Conformation-based refinement of 18-mer DNA structures
Source: Acta Crystallogr D Struct Biol. 2023 Jun 20;79(Pt 7):655–65. doi: 10.1107/S2059798323004679 (PMC10306069; doi:10.1107/S2059798323004679)
Supplement: Supplementary file 1 [file d-79-00655-sup1.pdf]

STRUCTURAL
BIOLOGY

Volume 79 (2023)

Supporting information for article:

Conformation-based refinement of 18-mer DNA structures

Jakub Svoboda, Daniel Berdár, Petr Kolenko, Jiří Černý, Zora Nováková, Jiří Pavlíček and Bohdan Schneider

.

Table S1. The final crystallization conditions for all nine newly reported 18-mers.

Central dinucleotide	PDB	Final condition
AC	7Z7L	10 mM KCl, 80 mM NaCl, 10 mM $SrCl_2$, 10 mM Spermine $(HCl)_4$, 25 % MPD, 40 mM Sodium Cacodylate (pH 6.5)
AG	7Z82	40 mM NaCl, 10 mM $SrCl_2$, 12 mM Spermine $(HCl)_4$, 22 % MPD, 40 mM Sodium Cacodylate (pH 6.5)
AT	7Z7K	30 mM NaCl, 20 mM $SrCl_2$, 12 mM Spermine $(HCl)_4$, 22 % MPD, 40 mM Sodium Cacodylate (pH 6.5)
CC	7Z7M	20 mM KCl, 60 mM NaCl, 10 mM $SrCl_2$, 10 mM Spermine $(HCl)_4$, 22 % MPD, 40 mM Sodium Cacodylate (pH 6.5)
CG	7Z7U	40 mM NaCl, 10 mM $SrCl_2$, 12 mM Spermine $(HCl)_4$, 25 % MPD, 40 mM Sodium Cacodylate (pH 6.5)
GC	7Z7W	5 mM KCl, 50 mM NaCl, 25 mM $SrCl_2$, 14 mM Spermine $(HCl)_4$, 20 % MPD, 40 mM Sodium Cacodylate (pH 6.5)
GT	7Z7Y	40 mM NaCl, 10 mM $SrCl_2$, 12 mM Spermine $(HCl)_4$, 20 % MPD, 40 mM Sodium Cacodylate (pH 6.5)
TA	7Z7Z	40 mM NaCl, 10 mM $SrCl_2$, 12 mM Spermine $(HCl)_4$, 18 % MPD, 40 mM Sodium Cacodylate (pH 6.5)
TC	7Z81	40 mM NaCl, 10 mM $SrCl_2$, 10 mM Spermine $(HCl)_4$, 18 % MPD, 40 mM Sodium Cacodylate (pH 6.5)

Table S2. Comparison of the refinement not using (labeled No restr) and using (Restr) NtC restraints for six structures Chom-18-TC, Chom-18-CG, Chom-18-AG, Chom-18-GC, Chom-18-GT and Chom-18-AT. Columns Δcon label change of the confal score and ΔRSCC of the RSCC between the restrained and non-restrained models.

	Chom-18-TC				Chom-18-CG				Chom-18-AG			
Step	No restr	Restr	Δcon	ΔRSCC	No restr	Restr	Δcon	ΔRSCC	No restr	Restr	Δcon	ΔRSCC
1-2	AA08	AA04	53	0.001	AA04	AA04	-9	-0.020	AA00	AA04	17	0.021
2-3	AA00	AA00	46	-0.005	AA00	AA00	3	-0.014	AA08	AA00	9	0.016
3-4	AA00	AA00	16	0.005	AB05	AA00	5	-0.031	AA08	AA00	18	0.007
4-5	AA10	AA00	32	0.023	NANT	AA00	82	-0.035	NANT	AA00	79	0.013
5-6	AA00	AA00	22	0.045	AA00	AA00	38	-0.014	AA04	AA00	7	0.012
6-7	AA01	AA01	9	0.097	AA04	AA01	17	-0.002	AA01	AA01	49	0.002
7-8	AA08	AA08	9	0.070	AA08	AA08	4	0.012	AA08	NANT	-9	0.010
8-9	AA00	AA00	50	0.011	AA08	AA11	-26	0.004	AA00	AB01	19	0.000
9-10	AA09	AA00	0	0.002	AA08	AA08	10	0.001	AA03	NANT	-27	0.018
10-11	AA10	NANT	-9	-0.001	AA10	AA00	47	0.051	NANT	NANT	0	0.009
11-12	NANT	NANT	0	-0.014	NANT	AA10	25	0.059	NANT	NANT	0	-0.042
12-13	AA08	AA08	-16	-0.020	AA08	AA08	-11	-0.011	NANT	AA08	56	-0.027
13-14	AA00	AA00	21	0.016	AA00	AA00	31	-0.020	AA00	AA00	20	-0.018
14-15	AA00	AA00	0	0.023	AA00	AA00	-26	-0.008	AA03	AA00	-3	-0.017
15-16	AA11	AA10	-9	0.010	AA01	AA10	-22	-0.018	AA06	AA10	-21	0.004
16-17	AA04	AA00	4	0.003	AA00	AA00	-2	-0.003	AA08	AA00	26	0.025
17-18	NANT	AB05	85	-0.002	AB05	AB05	-5	-0.012	NANT	AB05	86	0.037
Overall			18	0.014			9	0.011			18	0.065
Δ Rfree	-0.019				-0.020				-0.123			

	Chom-18-GC				Chom-18-GT				Chom-18-AT			
Step	No restr	Restr	Δcon	ΔRSCC	No restr	Restr	Δcon	ΔRSCC	No restr	Restr	Δcon	ΔRSCC
1-2	AA04	AA04	-1	0.006	AA04	AA04	1	0.011	AA04	AA04	-19	-0.024
2-3	AA00	AA00	1	0.007	AA00	AA00	-1	0.016	AA00	AA00	3	-0.002
3-4	AA00	AA00	-4	-0.003	AA00	AA00	0	0.007	AB05	AA00	62	-0.010
4-5	AA00	AA00	1	-0.008	AA00	AA00	6	-0.004	NANT	AA00	73	-0.007
5-6	AA00	AA00	3	0.003	AA00	AA00	6	0.001	AA00	AA00	5	-0.004
6-7	AA01	AA01	12	0.000	AA01	AA01	8	0.002	AA10	AA01	1	-0.012
7-8	AA08	AA08	15	-0.001	AA08	NANT	-79	-0.001	AA08	AA08	-11	-0.005
8-9	AA00	AA00	-31	0.003	AA00	AA00	2	0.004	AA00	AA00	-39	0.017
9-10	AA00	AA08	14	0.008	AA08	AA00	-8	0.000	AA03	AA03	33	0.053
10-11	NANT	AA00	32	0.008	NANT	AA06	29	-0.002	NANT	NANT	0	0.033
11-12	NANT	AA10	23	-0.004	NANT	AA11	66	0.011	NANT	AA01	34	0.002
12-13	AA08	AA08	0	0.001	AA08	AA08	1	0.011	AA03	AA08	3	-0.005
13-14	AA00	AA00	1	0.000	AA00	AA00	-2	0.002	AA04	AA00	17	-0.009
14-15	AA00	AA00	-3	-0.001	AA00	AA00	4	0.001	AA00	AA00	-2	0.011
15-16	AA01	AA10	-8	0.007	AA10	AA10	-1	0.000	AA10	AA10	-18	0.005
16-17	AA00	AA00	-1	0.002	AA00	AA00	11	-0.004	AA00	AA00	0	-0.001
17-18	AB05	AB05	-2	0.002	AB05	AB05	0	-0.001	AB05	AB05	21	0.007
Overall			3	0.008			2	0.010			10	0.028
Δ Rfree	-0.015				-0.012				-0.016			

Table S3. List of interatomic distances between neighboring helices shorter than 4 Å in 7Z7L. The packing distances are similar in all the other nine analyzed 18-mers.

Base1	Res1	Atom1	Base2	Res2	Atom2	Length [Å]
DG	1	N3	DG	5	N2	2.96
DG	1	N1	DG	6	O4'	3.06
DG	4	N2	DG	1	O4'	3.18
DC	15	O2	DG	1	C2'	3.33
DG	5	C2'	DG	1	N7	3.34
DG	7	C4'	DC	18	O3'	3.38
DG	5	N3	DG	1	N3	3.45
DG	5	C1'	DG	1	C8	3.54
DG	6	C4'	DG	1	C6	3.55
DG	7	O3'	DC	18	O3'	3.60
DG	6	C5'	DG	1	O6	3.60
DC	15	O3'	DG	2	C4'	3.61
DA	16	O5'	DG	2	C5'	3.63
DA	16	O4'	DG	1	O3'	3.63
DG	6	C1'	DG	1	N2	3.63
DC	15	OP1	DC	15	OP1	3.68
DG	5	N2	DG	1	N2	3.69
DC	15	C2'	DG	2	O4'	3.72
DG	6	O3'	DC	18	C5	3.73
DG	7	C5'	DC	18	O3'	3.76
DG	7	O5'	DC	18	C2'	3.79
DG	6	O3'	DC	18	N4	3.80
DA	16	C4'	DG	1	O3'	3.81
DC	15	C2'	DG	2	C4'	3.85
DA	16	C4'	DG	2	OP1	3.91
DC	18	C3'	DG	7	O5'	3.91
DG	6	C2'	DC	18	N3	3.92

Table S4. Full NtC assignments of the analyzed 18-mers of general sequences 5'-GGTGGGGC-XZ-GCCCCACC-3'.
More detailed analysis can be retrieved from the webservice dnatco.datmos.org.

-XZ-	AT	CG	GC	TA	CC	TC	TT	AC	GT	AG
PDB	7Z7K	7Z7U	7Z7W	7Z7Z	7Z7M	7Z81	6ROS	7Z7L	7Z7Y	7Z82
A_DG_1_DG_2	AA04	AA04	AA04	AA00	AA00	AA04	AA08	AA04	AA04	AA04
A_DG_2_DT_3	AA00	AA00	AA00	AA00	AA00	AA00	AA00	AA00	AA00	AA00
A_DT_3_DG_4	AA00	AA00	AA00	AA00	AA08	AA00	AA08	AA00	AA00	AA00
A_DG_4_DG_5	AA00	AA00	AA00	AA01	AA04	AA00	AA04	AA00	AA00	AA00
A_DG_5_DG_6	AA00	AA00	AA00	AA08	AA00	AA00	AA00	AA00	AA00	AA00
A_DG_6_DG_7	AA01	AA01	AA01	AA06	AA01	AA01	AA10	AA01	AA01	AA01
A_DG_7_DC_8	AA08	AA08	AA08	NANT	AA08	AA08	AA08	AA08	NANT	NANT
**A_DC_8_DX_9**	**AA00**	**AA11**	**AA00**	**AA08**	**AA00**	**AA00**	**AA00**	**AA00**	**AA00**	**AB01**
**A_DX_9_DY_10**	**AA03**	**AA08**	**AA08**	**NANT**	**AA00**	**AA00**	**AA08**	**AA00**	**AA00**	**NANT**
**A_DY_10_DG_11**	**NANT**	**AA00**	**AA00**	**AA00**	**NANT**	**NANT**	**NANT**	**AA06**	**AA06**	**NANT**
A_DG_11_DC_12	AA01	AA10	AA10	AA08	NANT	NANT	NANT	AA11	AA11	NANT
A_DC_12_DC_13	AA08	AA08	AA08	AA00	AA08	AA08	BA08	AA08	AA08	AA08
A_DC_13_DC_14	AA00	AA00	AA00	AA03	AA00	AA00	AA00	AA00	AA00	AA00
A_DC_14_DC_15	AA00	AA00	AA00	AA08	AA00	AA00	AA08	AA00	AA00	AA00
A_DC_15_DA_16	AA10	AA10	AA10	AA06	AA01	AA10	AA06	AA10	AA10	AA10
A_DA_16_DC_17	AA00	AA00	AA00	AA08	AA00	AA00	AA08	AA00	AA00	AA00
A_DC_17_DC_18	AB05	AB05	AB05	NANT	AB05	AB05	AB05	AB05	AB05	AB05

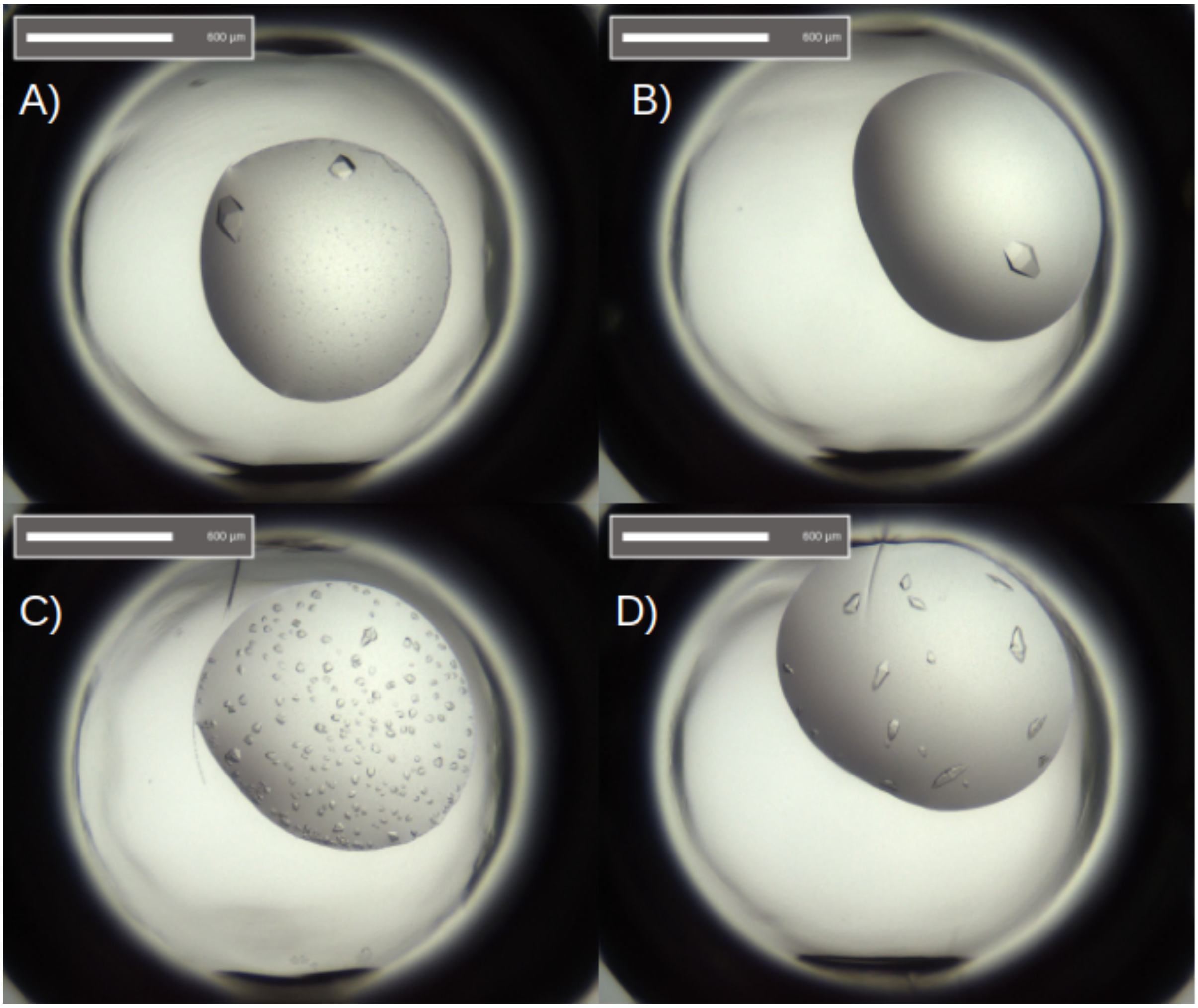

Figure S1. Microscope pictures from the crystallization hotel Formulatrix at the Centre of Molecular Structure in Institute of Biotechnology of the Czech Academy of Sciences. Parts A and B depicts tetragonal crystals of Chom-18-AC in condition containing 10 mM KCl, 60 mM NaCl, 10 mM $SrCl_2$, 12 mM Spermine $(HCl)_4$, 30 % MPD, 40 mM Sodium Cacodylate (pH 6.5). Parts C and D show crystals of Chom18-GT in condition containing 20 mM $SrCl_2$, 12 mM Spermine $(HCl)_4$, 20 % MPD, 40 mM Sodium Cacodylate (pH 6.5).

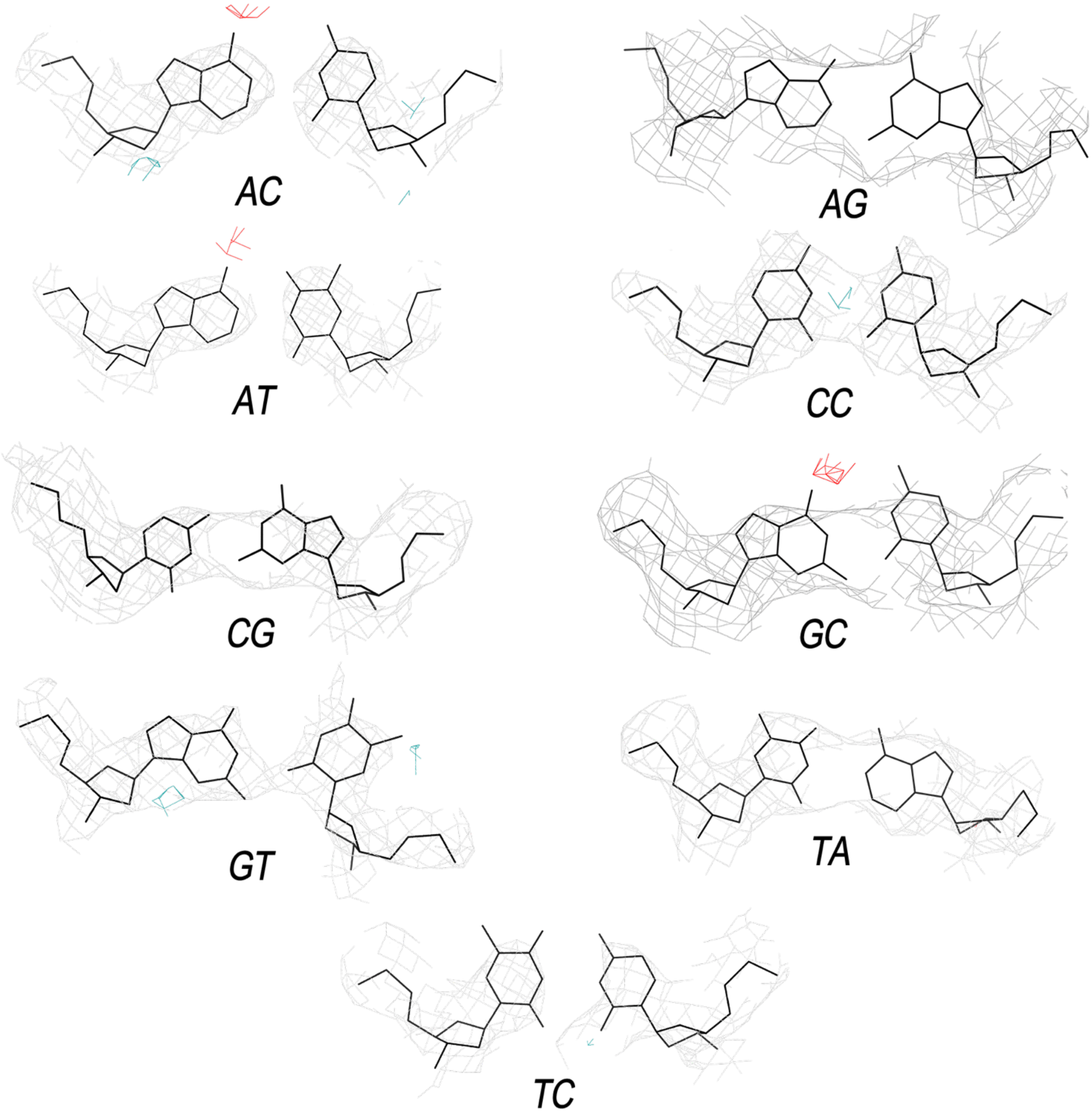

Figure S2: Electron density maps of central base pairs in the nine newly reported Chom-18-XZ variants. The 2mF_o–DF_c electron density is contoured in grey at 1σ level, the mF_o–DF_c electron density is contoured in green for positive and in red for negative at 3σ level. Images were drawn with CCP4MG (McNicholas et al. 2011).

Circular Dichroism - structural dispersity in solution

Even sequences fully compatible with Watson-Crick pairing can switch to short-lived and low-populated species in the Hoogsteen pairing mode (Hoogsteen 1963) (Nikolova, Kim et al. 2011) or even form stable duplexes with non-Watson-Crick pairs (Kim, Nikolov et al. 1993).DNA structural plasticity is observed in solution by spectral methods such as circular dichroism spectroscopy and nuclear magnetic resonance (Vorlickova, Kejnovska et al. 2012) (Del Villar-Guerra, Trent et al. 2018). NMR studies suggest that G-rich sequences form guanine tetrads in solution while crystal structure of the same sequence accommodates duplex with isolated G-G pairs (Skelly, Edwards et al. 1993). Additionally, spectral, and thermal analysis of palindromic sequences suggest the existence of a mixture of hairpin and duplex with dynamic equilibrium (Jaumot, Escaja et al. 2002).

Circular dichroism measurements. Oligonucleotides purchased from Sigma Aldrich were dissolved in 50 mM Tris pH 8 to final concentration of 20 μM. Prior to the experiment stocks containing DNA were annealed by heating up to 95 ˚C for 5 minutes followed by cooling down at room temperature. Spectra were recorded on Chirascan-plus spectrophotometer (Applied Photophysics, Leatherhead, UK) over wavelength range 205-350 nm and steps of 1 nm. The final spectra were normalized by DNA concentration to yield molar ellipticities.

Results. As in our previous studies of REP-related oligonucleotides (Charnavets, Nunvar et al. 2015) (Kolenko, Svoboda et al. 2020), we investigated DNA behavior in solution by circular dichroism. Their spectra (Figure S3) were collected as described in supplementary material and (Kolenko, Svoboda et al. 2020). The spectra of all ten analyzed 18-mers show complex sequence-dependent features. The A or B right-handed helical forms are most likely present in solution of all analyzed 18-mers as their CD spectra show features typical for these DNA forms - negative peaks at 210 and 238 nm and positive peak or saddle at 275 nm. (Vorlickova, Kejnovska et al. 2012). However, the spectra carry information of presence of other forms. The oligonucleotides unable to form Watson-Crick pairs in the center, namely Chom-18-TT, -TC, -CC, -GT and to some extent -TA, display a large positive peak at 285 nm and negative peak at 237 nm indicating at least partial formation of a G-tetraplex but they miss a distinctive negative band at 260 nm and a positive band around 215 nm, typical for all G-tetraplex spectra; Chom-18-TA spectrum is similar. In addition, the Chom-18-GC, -AC, -CG, -AT spectra show a saddle between 270 and 290 nm not observed in double helical structures. In summary, the CD spectra confirm our previous observations of conformational heterogeneity of REP-related oligonucleotides in solution but regardless of the sequence of the central dinucleotide, they all crystallize as A-form double helices.

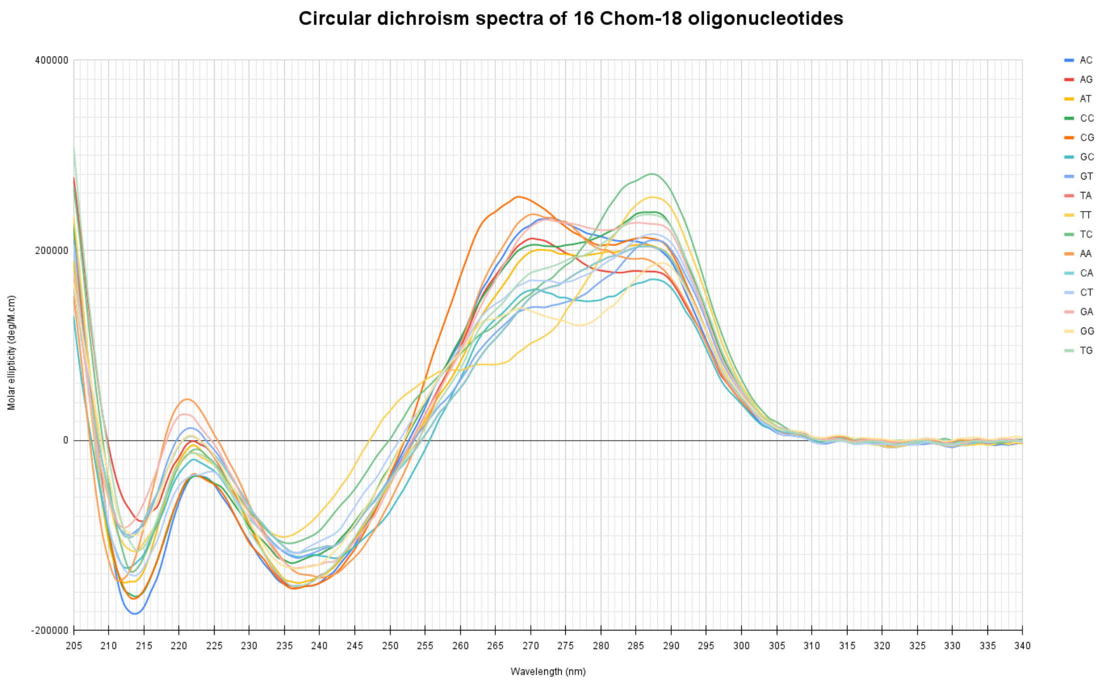

Figure S3. Circular dichroism spectra of 16 18-mers of sequence 5'-GGTGGGGC-XZ-GCCCCACC-3'. Recorded signals illustrate the complicated nature of conformational space in the solution.

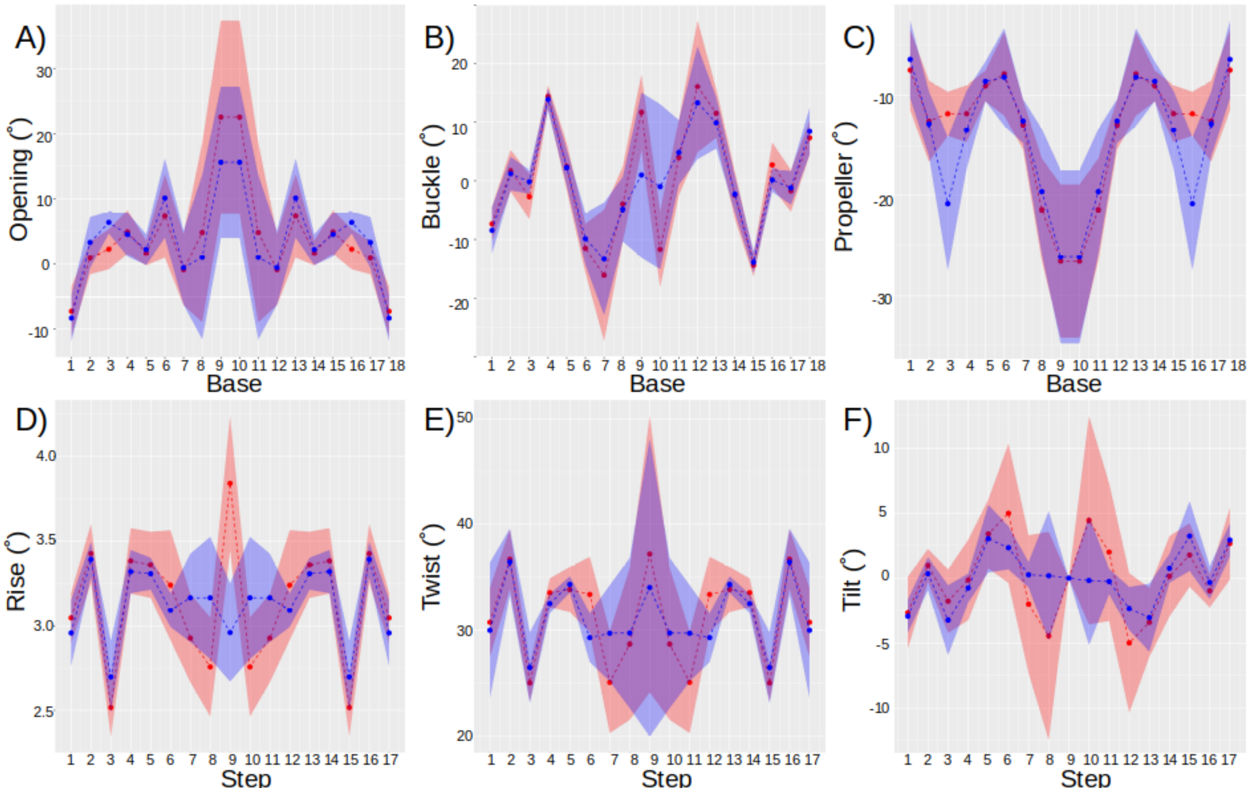

Figure S4. Graphs showing variability of base pair and step parameters Opening (a), Buckle (b), Propeller (c), Rise (d), Twist (e) and Tilt (f) for ten analyzed structures. Dashed lines represent the average values, and the widths of ribbon are the standard deviations. Parameters for CBPs are depicted in blue and for non-CBPs in red. Manually calculated in the DSSR (Li, Olson et al. 2019).

We analyzed base and helical parameters such as opening, buckle, propeller, rise, twist, and tilt (Neidle 2008) of the reported structures. Parameters describing the central base pairs are missing in the PDB-processed flat files of three structures (7Z7L - AC, 7Z7M - CC, 7Z7W - GC), their values were therefore calculated in the web DSSR (Li, Olson et al. 2019). Most parameters show the largest variability at the central region of the duplex, exceptions are rise and tilt (supplementary Figure S4), but we do not observe any clear trends discriminating the structures with Watson-Crick and non-Watson-Crick pairing.

References

Del Villar-Guerra, R., Trent, J. O. & Chaires, J. B. (2018). *Angew. Chem. Int. Ed. Engl.* **57**, 7171-7175.

Hoogsteen, K. (1963). *Acta Cryst.* **16**, 907-916.

Jaumot, J., Escaja, N., Gargallo, R., González, C., Pedroso, E. & Tauler, R. (2002). *Nucleic Acids Res.* **30**, e92-e92.

Kim, J. L., Nikolov, D. B. & Burley, S. K. (1993). *Nature* **365**, 520-527.

Li, S., Olson, W. K. & Lu, X. J. (2019). *Nucleic Acids Res.* **47**, W26-W34.

McNicholas, S., Potterton, E., Wilson, K. S. & Noble, M. E. M. (2011). Acta Cryst. D67, 386-394.

Neidle, S. (2008). Principles of Nucleic Acid Structure. London, Academic Press.

Nikolova, E. N., Kim, E., Wise, A. A., O'Brien, P. J., Andricioaei, I. & Al-Hashimi, H. M. (2011). *Nature* **470**, 498-502.

Pettersen, E. F., Goddard, T. D., Huang, C. C., Meng, E. C., Couch, G. S., Croll, T. I., Morris, J. H. & Ferrin, T. E. (2021). *Protein Sci.* **30**, 70-82.

Skelly, J. V., Edwards, K. J., Jenkins, T. C. & Neidle, S. (1993). *Proc. Natl. Acad. Sci. USA* **90**, 804-808.

Vorlickova, M., Kejnovska, I., Bednářová, K., Renciuk, D. & Kypr, J. (2012). *Chirality* **24**, 691-698.
